# Photosensitized Methylene Blue Nanoparticles: A Promising Approach for the Control of Oral Infections

**DOI:** 10.3390/biomedicines13030673

**Published:** 2025-03-10

**Authors:** Magali Parizzi, Aline Rosa Almeida, Gabriel Salvador, Diogo Dominguini, Mylena Fernandes, Daniela Becker, Michael Ramos Nunes, Anelise Viapiana Masiero, Cleonice Gonçalves da Rosa

**Affiliations:** 1 Multi-User Laboratory, Graduate Program in Environment and Health, Planalto Catarinense University, Lages 88509-900, SC, Brazil; maga@uniplaclages.edu.br (M.P.); mylena.fernandes@gmail.com (M.F.); anemasiero@uniplaclages.edu.br (A.V.M.); 2 Laboratory of Plasmas, Films, and Surfaces, Santa Catarina State University (UDESC), Joinville 89219-710, SC, Brazil; alinerosaufpr@gmail.com (A.R.A.); daniela.becker@udesc.br (D.B.); 3 Federal Institute of Santa Catarina, Lages 88506-400, SC, Brazil; salvagabriel@gmail.com (G.S.); michael.nunes@ifsc.edu.br (M.R.N.); 4 Laboratory of Experimental Pathophysiology, Graduate Program in Health Sciences, University of Southern Santa Catarina (UNESC), Criciúma 88806-000, SC, Brazil; diogo_dominguini@unesc.net; 5 Department of Endodontics, College of Dentistry and Dental Clinics, University of Iowa, Iowa City, IA 52242, USA

**Keywords:** nanotechnology, controlled release, antimicrobial resistance, biocompatibility

## Abstract

**Introduction:** Oral infections pose significant public health challenges, often exacerbating other comorbidities and increasing systemic health risks. Traditional treatments often fail to eliminate persistent micro-organisms and contribute to the rise of antimicrobial resistance. Nanoparticulate systems offer a promising solution by delivering active agents directly to targeted sites, providing more effective and localized treatment options. **Objective:** This study aimed to synthesize and characterize methylcellulose nanoparticles containing methylene blue at different concentrations using the nanoprecipitation method. We also evaluated their biocompatibility and antimicrobial activity against key micro-organisms commonly found in oral infections. **Methods:** The study involved physicochemical and morphological characterizations, including encapsulation efficiency, particle size, polydispersity index, zeta potential, and transmission electron microscopy (TEM). Additionally, controlled release profiles, antimicrobial efficacy against major oral pathogens, and biocompatibility in vitro assessments were performed. **Results:** The results revealed encapsulation efficiency between 99.1 and 98.8%, with particle sizes ranging from 186 to 274 nm and a zeta potential of 1.7 to 2.9 mV achieved at lower concentrations of methylene blue and methylcellulose. The nanoparticles demonstrated sustained drug release of 85% for the smaller particles and 45% for the larger particles for more than 10 h. The nanoparticles exhibited superior antimicrobial activity compared to pure methylene blue. Cell viability studies indicated that the nanoparticles were biocompatible with approximately 40% cell viability at lower concentrations of the nanoparticles. **Conclusions:** These findings suggest that methylene blue nanoparticles could serve as a promising adjunct in dental treatments. They offer targeted antimicrobial action while potentially reducing the development of antimicrobial resistance.

## 1. Introduction

Throughout life, humans are often infected by various micro-organisms, including bacteria, fungi, yeasts, and viruses. The oral cavity hosts a wide range of micro-organisms that colonize the surface of the teeth, periodontium, and soft tissues, maintaining a dynamic balance with the environment. However, when this balance is disrupted, resulting in a condition known as dysbiosis, diseases such as caries and periodontal disease can develop [1,2]. In more severe cases, oral pathogenic biofilms can contribute to the onset of systemic [3] diseases. Several factors can contribute to dysbiosis, including changes in the composition of saliva, systemic conditions and, notably, excessive sucrose consumption combined with poor oral hygiene [1]. Highly acidic and acid-resistant species associated with oral problems include *Streptococcus mutans*, *Lactobacillus*, *Actinomyces*, *Bifidobacterium*, and *Scardovia species* [4,5].

In terms of infectious processes, it is noteworthy to highlight that human pathogens have become progressively more resistant to antimicrobial treatments, posing an increasing threat to health. Traditional antibiotics are becoming less effective, while bacterial populations are evolving rapidly, which requires new approaches to control bacteria and infections [6,7]. New features are being developed to complement conventional therapies and target delivery of antimicrobial agents. These systems are designed to release the agent in a controlled manner, maintain its concentration for a long period of time, and be biodegradable as well as biocompatible [1].

In this context, photodynamic therapy (PDT) combined with photosensitizing agents is an excellent option as it targets several biomolecular structures (proteins, lipids, and nucleic acids) within the specific pathogenic target. It also inhibits the development of bacterial resistance by acting at a specific location [8], which makes PDT an effective and safe way to combat bacteria, fungi, viruses, and protozoa, thus reducing virulence factors [9].

Consequently, PDT reduces treatment times and the risk of bacteremia [10]. The light source for PDT is obtained through LEDs (light-emitting diodes) or lasers (light amplification by stimulated emission of radiation), with low-intensity lasers being used for over 30 years and showing positive clinical effects in various studies [11,12,13]. Low-power lasers in PDT are recognized for their analgesic, biomodulator, and anti-inflammatory effects. In addition to its use in dentistry, PDT is applied in medical treatments for cancer, photo-rejuvenation, fungal lesions, skin diseases, and as an option for treatment in the early phase of a COVID-19 infection [6,14,15].

Among the various photosensitizers (PS) used in photodynamic therapy (PDT), methylene blue (MB) stands out due to its high selectivity as an antimicrobial agent against both gram-positive and gram-negative bacteria [5] and, when used in combination with PDT, methylene blue generates oxygen and photo-oxidation products capable of inducing cell death [6]. For example, the cytotoxic reactive oxygen species (ROS) can achieve specific cancer cell death, necroptosis, autophagy, and necrosis or tumor tissue damage [16,17]. Its antimicrobial action is attributed to the interaction with laser light and oxygen, leading to chemical reactions that release free radicals or singlet oxygen, ultimately killing micro-organisms [6]

In recent years, PDT has been combined with photosensitizers incorporated into nanosystems, enabling more effective delivery to areas that are difficult to reach with conventional therapies, such as in the treatment of dental caries, periodontal diseases, and endodontic infections [18,19]. They can also be used for translocating them into cellular compartments, thereby producing significant amounts of ROS in the target tissues. Nanoparticles and/or polymeric nanoparticles stand out among the nanoparticulate systems carrying antimicrobial agents [11,20]. Encapsulation technology consists of biologically loading active compounds, such as methylene blue, into polymer matrices such as methylcellulose, maintaining their physical–chemical, biological, controlled release, and enhanced antimicrobial action [2].

One widely used method for nanoencapsulation is nanoprecipitation, which is fast, simple, and enables the production of nanoparticles (100–300 nm) with a narrow and unimodal distribution [21]. This approach enhances the bioavailability and solubility of photosensitizing agents, protects them from external and physical–chemical factors within the polymeric matrix, and reduces bacterial resistance by prolonging agent retention at the treatment site. It also minimizes side effects, improving therapeutic efficacy [6,8,22].

The aim of this study was to synthesize and characterize methylene blue nanoparticles in methylcellulose matrices at different concentrations by the nanoprecipitation method and to evaluate their biocompatibility and antimicrobial action against micro-organisms frequently found in oral infections.

## 2. Material and Methods

### 2.1. Materials

The following materials were used in this study: methylene blue, methylcellulose, poloxamer 407 by Sigma-Aldrich (Saint Louis, MO, USA). The culture media Mueller-Hinton agar and Tryptic Soy agar (Himedia, Thane, India), Brain and Heart Infusion Agar (BHI) (Merck, Darmstadt, Germany), Sabourad agar (Oxoid, Basingstoke, UK), and the microorganisms *Aggregatibacter Actinomycetemcomitans*—ATCC 29522 (Plast Labor, Rio de Janeiro, Brazil), *S. mutans* ATCC-25175 and *Escherichia coli* LB 25922, *Staphylococcus aureus* LB 25923, *Pseudomonas aeruginosa* LB 27853, *Enterococcus faecalis* LB 29212 (Laborclin, São José do Rio Preto, Brazil), and *C. albicans* (Brasil Cientifica, Pinhais, Brazil). The remaining reagents used were purchased from Sigma-Aldrich.

### 2.2. Synthesis of Methylene Blue Nanoparticles

Methylene-blue-loaded methylcellulose nanoparticles were prepared using the nanoprecipitation method in triplicate (*n* = 3) [20,23,24]. First, 0.001 g and 0.005 g of MC were added to 10 mL of water. Then, 0.005% (*m*/*v*) of methylene blue was added to this solution. Next, 0.15 g of poloxamer 407 surfactant was added. Subsequently, 1 mL of these solutions was dispersed in 20 mL of ethanol (95%) and homogenized in an Ultra Turrax device at 10.000 rpm for 3 min. The control sample was synthesized without methylene blue.

To conduct the experiments, the nanoparticle samples were divided into four groups, as follows:

1. Nanoparticles with 0.001 g methylcellulose and 0.005% methylene blue (MC-0.001-MB 0.005%)
2. Nanoparticles with 0.005 g methylcellulose and 0.005% methylene blue (MC-0.005-MB 0.005%)
3. Nanoparticles with 0.001 g methylcellulose (MC 0.001-MB free)
4. Nanoparticles with 0.005 g methylcellulose (MC 0.005-MB free)

### 2.3. Physical–Chemical Characterization of Methylene Blue Nanoparticles

#### 2.3.1. Encapsulation Efficiency (EE)

The encapsulation efficiency of methylcellulose nanoparticles was evaluated in triplicate (*n* = 3) [25]. The nanoparticles underwent suspension centrifugation using Amicon Ultra Centrifugal filters with an Ultracel 30 k membrane. The procedure involved centrifuging the samples for 30 min at a speed of 6000 rpm to separate the encapsulated from the nonencapsulated compounds. The filtrate that passed through the filter membrane contained the nonencapsulated compound, which was then analyzed. Nonencapsulated methylene blue (supernatant) was quantified by UV-vis spectroscopy at a wavelength of 655 nm. The molar concentration of methylene blue was calculated using a calibration curve in an alcoholic solution of methylene blue. Encapsulation efficiency (EE) was calculated according to Equation (1):

(1) $$EE\;\left(\%\right)=\left(Initial\;MB-free\;MB\right)/\left(Initial\;MB\right)\times 100$$

#### 2.3.2. Determination of Particle Size (Z-Ave), Polydispersity Index (PDI), and Zeta Potential

The particle size (nm), polydispersity index, and zeta potential (mV) of nanoparticles were determined by dynamic light scattering (DLS) using a Zetasizer Nano Series device. Samples of methylene-blue-loaded methylcellulose nanoparticles and the control sample were accurately diluted with Milli-Q^®^ ultrapure water and measurements were performed in triplicate (*n* = 3) at 25 °C at an angle of 173°. For measurement purposes, the samples were placed in an electrophoresis cell.

#### 2.3.3. Morphological Analysis by Transmission Electron Microscopy

Nanoparticle morphology was evaluated by means of transmission electron microscopy (TEM) using a JEOL JEM-1011 microscope (JEOL, Tokyo, Japan) at 70 kV. Solutions containing methylene-blue-loaded methylcellulose nanoparticles and the control sample were previously diluted in ultrapure Milli-Q water and approximately 5 µL of each sample was deposited onto carbon-coated copper grids (200 mesh). After drying at room temperature, the grids were observed under the microscope. The obtained micrographs were analyzed using the Image J software version 1.54.

#### 2.3.4. Fourier Transform Infrared (FTIR)

The FTIR spectra of the nanoparticles produced were obtained between 4000 and 400 cm^−1^ using a Bruker FTIR model INVENIO-S with 32 scans and 4 cm^−1^ resolution. FTIR spectra were obtained using the ATR module.

#### 2.3.5. Profile Release Studies

Profile release studies on methylene blue nanoparticles were carried out using the same procedure as described in a previous study [26]. First, 5 mL of methylene blue nanoparticles were dispersed in 20 mL of phosphate buffer saline solution (PBS) (pH~7.4) at 37 ± 0.5 °C. Later, at predetermined time intervals (aliquots were removed hourly within the first 8 h and then every 12 h until the release curve reached a plateau), the methylene blue nanoparticle/buffer solution mixture was centrifuged and the supernatants were removed and replaced with the same volume of PBS buffer solution. The molar concentrations of methylene blue in the supernatant were determined based on the absorbance values measured at 655 nm and calculated by the calibration curve of methylene blue in PBS at pH∼7.4. The percentage of methylene blue released during a specific time interval was quantified using Equation (2).

(2) $$MB\;relesead\left(\%\right)=(MB\;relesead/MB\;initial)\times 100$$

where: [MB] released is the concentration of MB released in time (t) and [MB] initial is the concentration of MB being loaded into the methylcellulose nanoparticle.

### 2.4. Antimicrobial Evaluation of Methylene Blue Nanoparticles

The antimicrobial effectiveness of methylene blue nanoparticles was evaluated using two methodologies based on standard plate counting [27].

The analyzed samples were distributed into subgroups as described below:

1. Nanoparticles with 0.001 g methylcellulose with 0.005% methylene blue (MC-0.001-MB 0.005%)
2. Nanoparticles with 0.005 g methylcellulose with 0.005% methylene blue (MC-0.005-MB 0.005%)
3. Nanoparticles with 0.001 g methylcellulose (MC 0.001-MB free)
4. Nanoparticles with 0.005 g methylcellulose (MC 0.005-MB free)
5. Methylene blue (MB 0.005%)

For bacterial cell cultures for each strain of the micro-organisms *S. mutans*, *S. aureus*, *C. albicans*, *P. aeruginosa*, *E. faecalis*, and *E. coli*, a standardized suspension of 10^8^ cells·mL^−1^ of micro-organisms was developed in BHI broth in a plate at 37 °C. *Actinomyces actinomycetemcomitans* was incubated in TSB broth in an anaerobic jar at 37 °C for 72 h. All micro-organisms were standardized in saline solution by turbidity parameters using the McFairland scale.

To evaluate antimicrobial activity, the microbial suspension was combined with the photosensitized samples in a 96-well plate at a 1:3 ratio. Initially, the micro-organisms were in contact with the methylene blue nanoparticles and the control samples for 5 min (preirradiation period). Subsequently, the samples were irradiated using a low-power diode laser (DMC-therapy) operating at 660 nm, with continuous emission, an effective power of 100 mW, and a total energy of 9 joules applied over 90 s [27]. The laser tip was positioned 10 mm from the top of the wells and the irradiation was performed in an intercalated manner between groups of 4 wells, ensuring adequate distancing of the light source and preventing overlap between the samples.

Then, the treated suspensions (micro-organisms + samples) of each group were placed in a buffer solution (10^−1^) and decimals dilutions were performed; subsequently, the diluted samples were plated onto Muller Hinton agar and incubated at 37 °C for 48 h. For C. albicans, incubation was performed in Sabourad agar at 37 °C for 48 h. For *Actinomyces actinomycetemcomitans*, the samples were incubated in a double layer of TSB and Mueller Hinton broth in an anaerobic jar at 37 °C for 72 h. The results were expressed as colony forming unit (CFU) per mL (CFU·mL^−1^).

### 2.5. Cytotoxicity Test and Cell Viability

Rabbit oral mucosal surface cells were initially cultured in Dulbecco’s Modified Eagle Medium (DMEM) with high glucose content supplemented with 10% bovine fetal serum, 100 units·mL^−1^ of penicillin, and 100 mg·mL^−1^ of streptomycin. The cells were maintained in a humidified environment with 5% CO_2_/95% air moisture at 37 °C. Single doses of nanoparticles (MC-0.001-MB 0.005% and MC-0.005-MB 0.005%) were added at concentrations of 25, 50, 75, and 100 μg·mL^−1^. The samples were irradiated before being placed in contact with the cells. For this purpose, a low-power diode laser (DMC-therapy) with a wavelength of 660 nm, continuous emission, a power of 100 mW, and a total energy of 9 joules applied over 90 s was used [27]. The laser tip was positioned 10 mm from the top of the wells and the irradiation was carried out alternately in groups of 4 wells, ensuring the proper distancing of the light source and preventing overlap between the samples. Cell viability of the mucosal surface cells was determined using the MTT assay (0.5 mg·mL^−1^) and exclusion of tripane blue. The cells were grown at a density of 10,000 cells/well in 96-well plates. The cells were stained with 20 μL of MTT stock solution (5 mg·mL^−1^) for 4 h. After that, the cells were dissolved with DMSO and the optical density was determined at 490 nm.

To assess cell viability, the percentage of viable cells in comparison to the control was calculated using the absorbance values relative to the ratio of the cells exposed to the treatment (Abs. sample) and the cell-free culture medium (Abs. white), as shown in Equation (3) below.

(3) Cell viability (%)= (Abs. sample)/(Abs. white)×100

### 2.6. Data Analysis

The data were expressed in means and standard deviation of the measurements performed in triplicate. The results underwent analysis of variance and the comparison of the means was performed by the ANOVA and Tukey’s tests with a significance level of 5% using the software STATISTICA 7.0.

## 3. Results and Discussion

### 3.1. Encapsulation Efficiency

Encapsulation efficiency is a key parameter in nanosystems for biomedical applications. In this study, the encapsulated formulations showed an encapsulation efficiency rate close to 100%, with values of 99.1% for the formulation MC 0.001-MB 0.005 and 98.8% for MC 0.005-MB 0.005 (*p* < 0.05) as shown in Table 1. The slight reduction in encapsulation efficiency in the formulation with higher methylcellulose weight (0.005 g of MC) can be explained by the increased viscosity of the solution, which hinders the complete encapsulation of methylene blue.

The results for encapsulation efficiency can be attributed to the chemical compatibility between the coating polarity (methylcellulose) and the active compound (methylene blue) compared to partially hydrophobic coatings such as cellulose [7].

Other authors [22] reported encapsulation efficiency values ranging from 56% to 89% when encapsulating methylene blue—a polar compound—in cellulose matrices, which have a partially hydrophobic character. On the other hand, lipophilic active compounds encapsulated in polar coatings tend to present lower efficiency rates, usually around 70%, owing to lower chemical interaction with the coating material [25].

This difference may be due to factors such as low affinity between the coating and the active compound, differences in the chemical nature and the polarity of the materials, and experimental conditions like the proportions between the polymeric matrix and the active compound [21].

The methylcellulose matrix ensures the stability of the active compound in encapsulation and enables a controlled release; thus, it is an alternative for therapeutic applications, for example, the treatment of diseases of the oral mucosa, such as oral lesions, the control of microbial infections, and tissue regeneration in patients with periodontal diseases [27].

### 3.2. Particle Size, Polydispersity Index, and Zeta Potential

**Figure 1:**
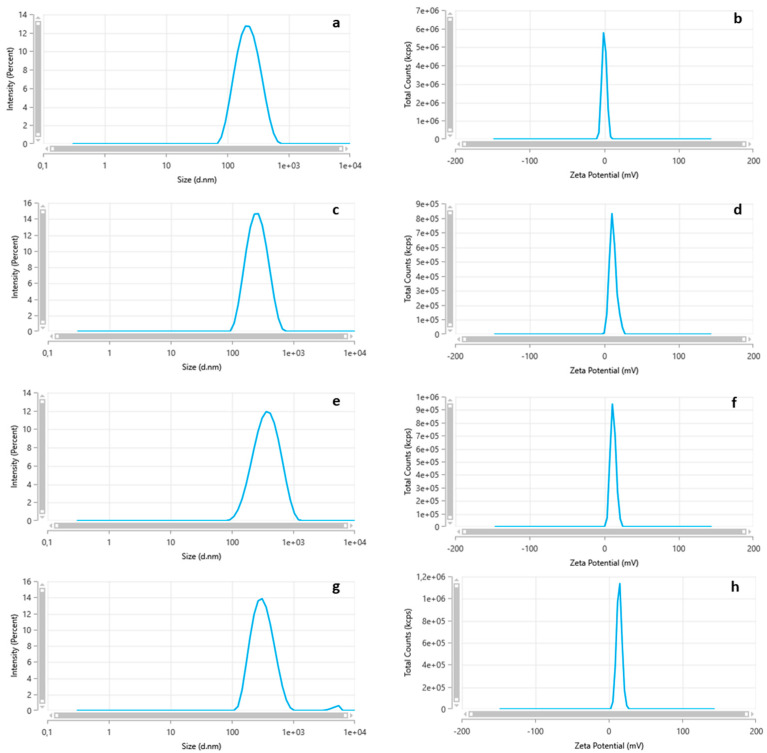
Particle size distribution and zeta potential. (**a**,**b**) MC 0.001-MB 0.005; (**c**,**d**) MC 0.005-MB 0.005; (**e**,**f**) MC 0.001-MB free and (**g**,**h**) MC 0.005-MB free.

Average particle size ranged between encapsulated and MB-free formulations. The formulation of MC 0.001-MB 0.005 showed smaller particles (186.2 ± 3.1 nm) and more homogeneous distribution (PDI = 0.274 ± 0.110), while the MC 0.005-MB 0.005 sample resulted in larger particles (274.1 ± 10.8 nm) and a slight increase in PDI (0.353 ± 0.003). The MB-free formulations exhibited larger particles and a more heterogeneous distribution, with sizes of 495.1 nm and high PDI (0.730 ± 0.007 for MC 0.001-MB free), as shown in Table 1 and Figure 1

Particle size control affects the physicochemical properties of the system, such as dispersion stability and encapsulation efficiency of methylene blue [20]. Factors that influence particle size include the nature and concentration of the polymer and surfactant in the organic phase, solvent polarity, proportion of the internal and external phases, and encapsulation efficiency [20]

The literature suggests that low concentrations of methylcellulose, like those used in this study, can produce formulations with lower size distributions [20]. Additionally, the proportion of internal to external phases is another important factor influencing particle size. This study used a ratio of 1:20, corroborating the findings of studies by other authors, who reported similar results for particle size in these proportions [20].

The methylene blue concentrations used in this study for nanoparticle formulations were lower than standard clinical recommendations in dentistry. This approach can reduce the risk of tooth staining, a common concern in clinical applications involving dyes. Previous studies have shown that they can ultimately stain tooth structure, potentially compromising aesthetics [28,29]. Methylene blue is a cationic compound that can bind to and interact with anionic structures, such as the phosphate atoms in the hydroxyapatite crystal potentially altering the phosphate/calcium ratio of the carbonate complex in root dentin [30]. This reaction between the phosphate and photosensitizer increases porosity, facilitating the penetration of methylene blue into the dentinal tubules [30].

Regarding the polydispersity index, the nanoparticles exhibited a homogeneous and unimodal size distribution, with most particles showing similar diameters and a single peak distribution. The polydispersity index of nanoparticles (approximately 0.3) is classified as low, indicating uniform particle size. This characteristic is highly desirable in nanoparticles, particularly in biomedical applications, as it ensures stability and uniformity of colloidal dispersion and minimizes agglomerations or precipitations [31].

In comparison, a study [32] encapsulated methylene blue in poly(lactic-co-glycolic acid, PLGA) matrices, resulting in nanoparticles with an average size of 166 nm and a polydispersity index of 0.287. These values are similar to those observed in this study, highlighting the reproducibility of the results across different encapsulation systems. The zeta potential directly relates to the electrical charge on the surface of suspended particles and is used to evaluate the stability of dispersions. In this study, the zeta potential values ranged from 0.6 to 4.6 mV for methylene-blue-loaded samples and from 1.4 to 6.6 mV for empty samples. These values suggest moderate stability of the system and higher potentials are usually associated with higher electrostatic stability [26].

For stabilized systems with cationic surfactants, zeta potential values greater than ±30 mV are expected, which help prevent agglomeration. In this study, the use of anionic surfactants, such as Polaxamer, resulted in physical–chemical stability induced by steric hindrance [25].

In this study, the loaded nanoparticles were stored under refrigeration (5 ± 2 °C) for three years and then evaluated for their physicochemical stability, considering the particle size and zeta potential. It was observed that both parameters underwent slight modifications: the particle size values were 190 nm and 295 nm for the formulations MC 0.001-MB 0.005 and MC 0.005-MB 0.005, respectively, and the zeta potential remained around +1.0 mV.

The results of this study highlight the factors that influence the size, distribution, and colloidal stability of methylene-blue-loaded nanoparticles. The analyses provide valuable insights for optimizing the physicochemical characterization of these nanosystems, with the aim of enhancing their therapeutic properties for biomedical applications.

### 3.3. Morphological Evaluation by TEM

**Figure 2:**
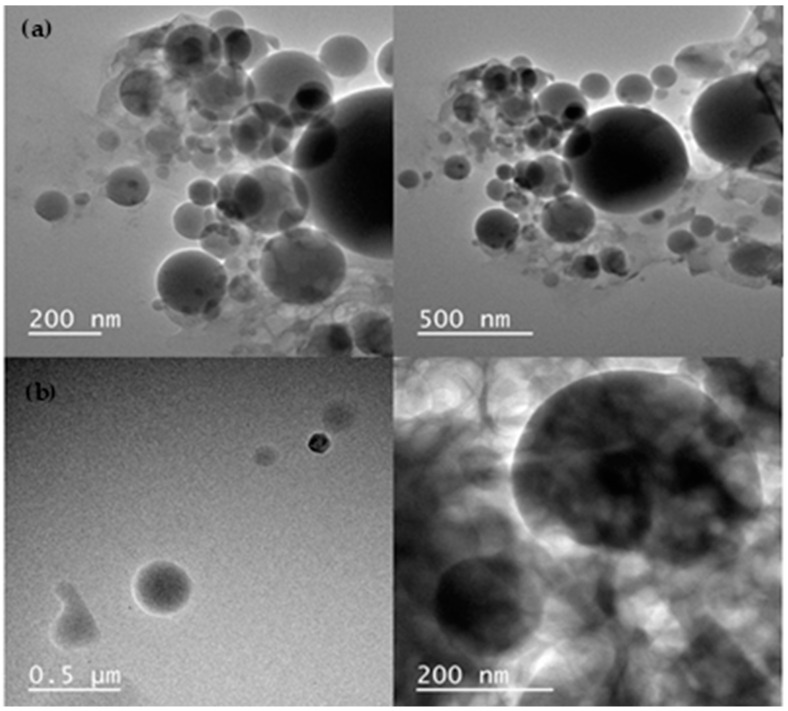
Micrographs of transmission electron microscopy. (**a**) MC0.001-MB 0.005 and (**b**) MC0.005-MB 0.005.

The morphology results are presented in the micrographs obtained by transmission electron microscopy (TEM) of formulations MC 0.001-MB 0.005 (Figure 2a) and MC 0.005-MB 0.005 (Figure 2b), which reveal distinct structural characteristics between the samples. Both formulations demonstrated smooth, compact, and spherical surfaces, with no visible free material or detectable pores—characteristics that are desirable for ensuring the integrity of the nanoparticles and protecting the encapsulated agent. Micrograph Figure 2a, corresponding to the formulation MC 0.001-MB 0.005, shows particles with a smaller diameter and a more homogeneous distribution, which favors the increase of the surface area. In contrast, micrograph Figure 2b, referring to the formulation MC 0.005-MB 0.005, shows particles with a larger diameter and less homogeneous distribution and a reduced surface area.

The mean nanoparticle diameter, determined by TEM, was approximately 100 nm. However, variations were observed between the particle sizes measured by TEM and dynamic light scattering (DLS) [31]. These discrepancies can be attributed to the preparation steps for TEM samples, such as solvent evaporation, which can alter the shape and size of the particles [26]. These observations highlight the importance of considering the specificities of each analytical technique and the methods used for sample preparation when interpreting results, as each method has distinct characteristics and limitations that can impact nanoparticle analysis.

### 3.4. Fourier Transform Infrared (FTIR)

**Figure 3:**
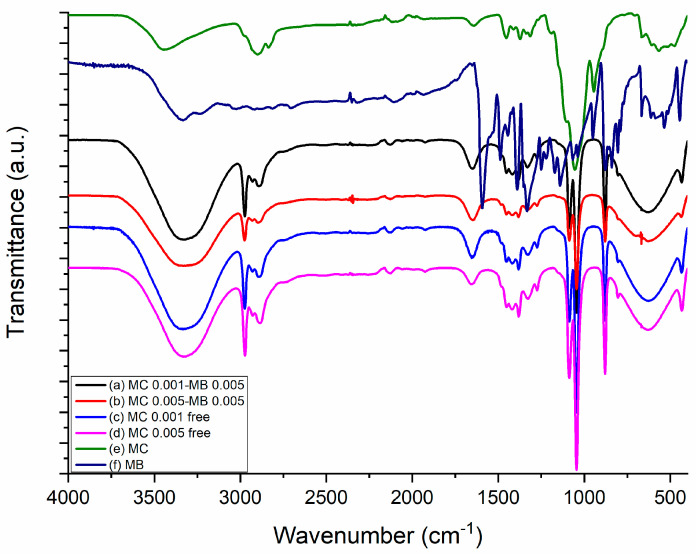
FTIR spectrum of nanoparticles. (a) MC 0.001-MB 0.005, (b) MC 0.005-MB 0.005, (c) MC 0.001-MB free, (d) MC 0.005-MB free, (e) MC and (f) MB.

Fourier transform infrared spectroscopy (FTIR) was employed to characterize the functional groups present in the samples of pure methylcellulose, pure methylene blue, and empty methylcellulose nanoparticles in Figure 3. The obtained spectrum revealed characteristic absorption bands of each compound, enabling the identification of chemical interactions within the nanoparticle structure.

In the spectrum of pure methylcellulose (Figure 3e), a broad band at ~3400 cm^−1^ was observed, attributed to the stretching vibration of the O-H bond, characteristic of the polymeric structure of cellulose and free hydroxyl groups. Additionally, a band at ~2900 cm^−1^ was assigned to the stretching vibration of the C-H bond in methyl (-CH_3_) groups, while the band with the maximum absorption at ~1650 cm^−1^ was associated with the deformation of adsorbed water in the structure. The presence of bands at ~1430 cm^−1^ and ~1375 cm^−1^ indicates C-H deformation vibrations and symmetric deformation of methyl groups, respectively. In the range of 1050–1150 cm^−1^, bands related to the stretching of C-O-C bonds, corresponding to the glycosidic structure of methylcellulose, were observed [33].

For pure methylene blue (Figure 3f), the FTIR spectrum presented significant bands associated with its molecular structure. The band with maximum absorption at ~1600 cm^−1^ is characteristic of the stretching of the C=C bond present in the aromatic ring, while the signal at ~1500 cm^−1^ indicates vibrations of the substituted aromatic ring. Additionally, bands at ~1400 cm^−1^ and ~1300 cm^−1^ were attributed to C-N and C=S bonds, respectively, while absorptions between 1200–1100 cm^−1^ correspond to the stretching of C-S-C bonds in methylene blue [34].

The FTIR spectrum of methylene-blue-loaded methylcellulose nanoparticles (Figure 3a,b) displayed characteristic absorption bands of both methylcellulose and methylene blue, indicating successful nanoparticle formation and possible interactions between the polymeric matrix and the encapsulated compound.

In the spectrum of MC 0.001-MB 0.005 (Figure 3a) and MC 0.005-MB 0.005 (Figure 3b), the broad band at ~3400 cm^−1^, attributed to O-H stretching vibrations, exhibited a shift and slight decrease in intensity compared to pure methylcellulose. This variation suggests hydrogen bonding interactions between the polymeric hydroxyl groups and methylene blue molecules. The characteristic C-H stretching band at ~2900 cm^−1^ remained present, confirming the integrity of the methylcellulose backbone after encapsulation.

Additionally, absorption bands in the region of 1600–1500 cm^−1^, corresponding to C=C stretching vibrations in the aromatic rings of methylene blue, were identified. The presence of bands at ~1400 cm^−1^ and ~1300 cm^−1^, attributed to C-N and C=S bonds, respectively, suggests molecular interactions between methylene blue and methylcellulose, possibly through electrostatic or van der Waals forces. The region between 1050–1150 cm^−1^, corresponding to C-O-C stretching vibrations of glycosidic bonds, remained evident in both spectra, indicating that the polymeric structure of methylcellulose was preserved after nanoparticle formation.

The spectrum analysis of empty methylcellulose nanoparticles (Figure 3c,d) revealed patterns similar to those of pure methylcellulose, with slight shifts in absorption bands, suggesting structural modifications resulting from nanostructuring. The O-H stretching, recorded at ~3400 cm^−1^, showed a slight variation in intensity, which may be attributed to the reorganization of the polymeric matrix. Additionally, the bands corresponding to C-O-C (1050–1150 cm^−1^) and C-H (2900 cm^−1^) bonds remained present, confirming the methylcellulose structure after nanoparticle formation.

### 3.5. Nanoparticle Release Profile

**Figure 4:**
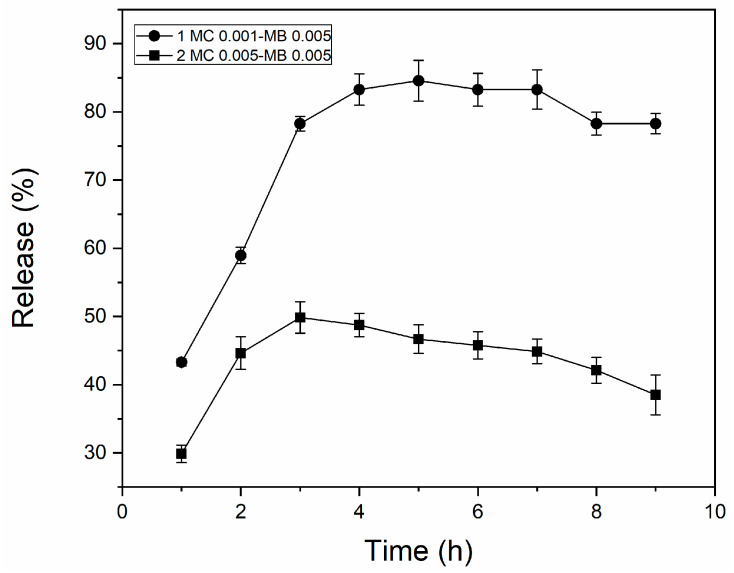
Profile release of methylene blue in methylcellulose matrices.

Figure 4 demonstrates that the nanoparticles exhibited a gradual release profile over 10 h. This behavior corresponds to a sustained release, where the release is extended over time without an active control mechanism. The formulation MC 0.001-MB 0.005, with smaller particles (186.2 ± 3.1 nm), showed a faster release, reaching about 85% of methylene blue in the first 10 h, and stabilization occurred between 4 and 6 h. This behavior can be attributed to the smaller particle size, which increases the surface area, facilitating the diffusion of the drug through the methylcellulose matrix.

In contrast, the formulation MC 0.005-MB 0.005, with larger particles (274.1 ± 10.8 nm), displayed a slower release, reaching approximately 45% of methylene blue in the period of analysis. The larger size of the particles reduces the available surface area, impeding drug diffusion and leading to a more gradual release, thereby preventing burst effects (rapid and uncontrolled initial release).

The positive interaction between methylene blue and methylcellulose enhanced the controlled release profile, showing that it is an effective coating that enables gradual drug release. Release kinetics are influenced by factors such as the concentration of the polymer matrix, the proportion of the active compound, the size and distribution of the nanoparticles, and the physicochemical characteristics of the system [20].

The formulations presented an adequate release profile for dental therapeutic systems, for example, in the treatment of caries, periodontal diseases, endodontic infection, and peri-implantitis. This behavior maintains therapeutic drug concentrations over extended periods, reduces the risk of adverse effects associated with peak releases, and promotes better adherence to treatment.

### 3.6. Cytotoxicity Test and Cell Viability Results

**Figure 5:**
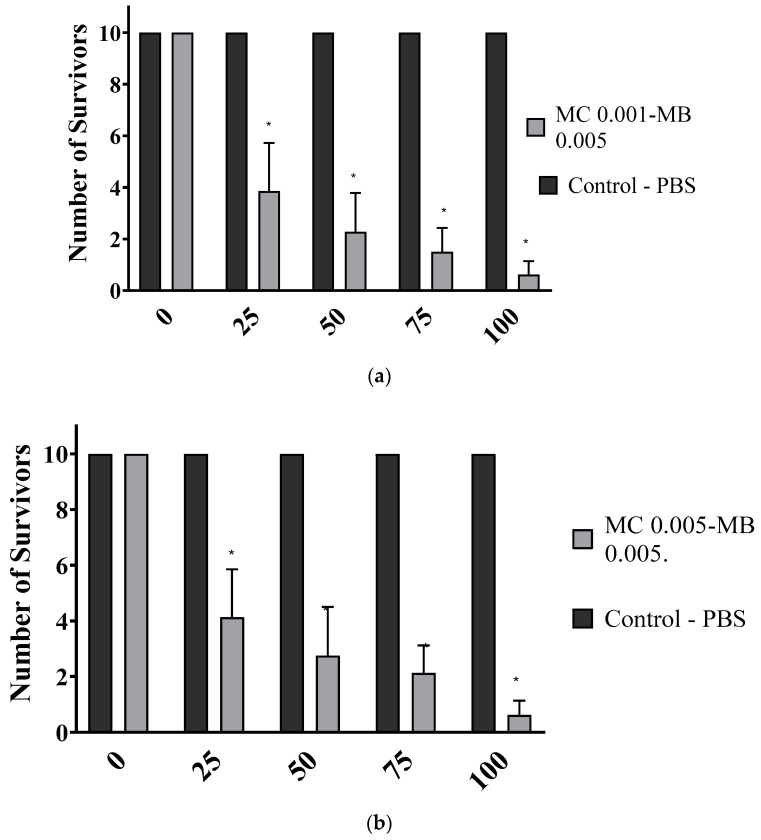
Survival of oral mucosa cells against different concentrations of methylene-blue-loaded methylcellulose nanoparticles. (**a**) MC 0.001-MB 0.005 and (**b**) MC 0.005-MB 0.005. The *p* values: * *p* < 0.05.

Figure 5 shows the cell viability results of methylene-blue-loaded methylcellulose nanoparticles. Figure 5a (MC 0.001-MB 0.005) shows that the concentration of 25% nanoparticles resulted in cell survival close to 40%, while higher concentrations of nanoparticles, between 75% and 100%, caused a significant reduction in cell viability. Figure 5b (MC 0.005-MB) shows a similar pattern, with a progressive reduction in cell viability at increasing concentrations, with the lowest survival rates recorded at 75% and 100%.

Although toxicity testing using cultured cells is a reliable and safe alternative that does not require the use of animals in experiments, the literature reports numerous factors that can interfere with the results and need to be taken into consideration [35]. The compound concentration might not have been optimal for the cell model used and the exposure time could also have influenced the results. Additionally, the methodology for assessing viability, the specific characteristics of the cell line tested, and the experimental conditions, such as temperature and pH, may have affected the data. These points will be taken into account to optimize the dosing and exposure conditions, ensuring the safety and efficacy of the compound in future clinical applications.

These results suggest that at low concentrations (up to 25%), nanoparticles can be used locally in the oral mucosa without causing cytotoxic effects or compromising the cellular viability of healthy tissue. This makes them a feasible alternative for therapeutic strategies for oral conditions.

### 3.7. Microbiological Evaluation

Methylene-blue-loaded methylcellulose nanoparticles demonstrated antimicrobial activity against *S. mutans*, *S. aureus*, *P. aeruginosa*, *E. coli*, *A. actinomycetemcomitans*, *E. faecalis*, and *C. albicans*, with variable effectiveness depending on the formulation being used. Associated with photodynamic therapy (PDT), these nanoparticles presented better results than pure methylene blue, as shown in Table 2.

The antimicrobial mechanism of methylene blue is based on its function as an exogenous photosensitizer during PDT. When irradiated with specific-wavelength laser light, methylene blue transfers energy to the molecular oxygen present in the tissues, generating reactive oxygen species (ROS), such as singlet oxygen and free radicals. These ROS cause oxidative damage to cell membranes, proteins, and the genetic material of micro-organisms, ultimately destroying them [5,6]. When on a nanometric scale, methylene blue loaded into methylcellulose nanoparticles, presents sustained release, potentiating its therapeutic and antimicrobial action.

The formulation MC 0.001-MB 0.005 was effective against *S. mutans* (3.5 × 10^3^ ± 7.1 × 10^2^ CFU·mL^−1^) and *E. faecalis* (5 × 10^2^ ± 7.1 × 10^2^ CFU·mL^−1^) while the growth of micro-organisms such as *P. aeruginosa*, *E. coli*, *and C. albicans* was inhibited below the detection limit (<10^2^ CFU·mL^−1^). MC 0.005-MB 0.005 demonstrated greater effectiveness against *S. mutans* (*A. actinomycetemcomitans* 5 × 10^2^ ± 7.1 × 10^2^ UFC/mL) and *S. aureus* (1.5 × 10^3^ ± 7.1 × 10^2^ CFU·mL^−1^) and it showed low viability for *P. aeruginosa* (5 × 10^2^ ± 7.1 × 10^2^ CFU·mL^−1^).

On the other hand, formulations with free methylene blue (MC 0.001-MB Free and MC 0.005-MB Free) showed lower antimicrobial activity. MC 0.001-MB Free was less effective against *P. aeruginosa* (7 × 10^3^ ± 2.8 × 10^3^ CFU·mL^−1^) and *S. mutans* (1.5 × 10^3^ ± 7.1 × 10^2^ CFU·mL^−1^) while MC 2.5-MB Free allowed higher growth of *A. actinomycetemcomitans* (2.5 × 10^4^ ± 3.5 × 10^3^ CFU·mL^−1^) and *E. coli* (5 × 10^5^ ± 7.1 × 10^5^ CFU·mL^−1^). Pure methylene blue (MB 0.005) presented the highest CFU/mL counts for all the study micro-organisms, especially *E. coli* (2.2 × 10^8^ ± 3 × 10^8^ CFU·mL^−1^) and *S. aureus* (26.8 × 10^5^ ± 2.5 × 10^5^ CFU·mL^−1^) and they showed lower effectiveness when nonencapsulated.

The nanometric size of methylcellulose particles facilitates penetration into bacterial biofilms and micro-organisms, altering the permeability of the cell membrane, which results in the loss of intracellular components essential for bacterial survival [36]. Even empty nanoparticles, without methylene blue loading, demonstrated antimicrobial activity from the rupture of cell membranes due to their small size. Some of these nanoparticles also exhibited bacteriostatic action, inhibiting microbial proliferation. The formulation MC 0.005-MB 0.005 stood out for its effectiveness against *S. mutans* and *P. aeruginosa*, while MC 0.001-MB 0.005 was more efficient against *E. faecalis*. This suggests that methylene blue concentration and the characteristics of nanoparticles could optimize antimicrobial activity against different micro-organisms.

Different formulations of methylene-blue-loaded nanoparticles associated with PDT have been tested both in vitro, showing promising results against the major micro-organisms related to oral biofilms optimizing the treatment of microbial infections [37,38]. The combination of PDT with nanoparticulate systems increases the antimicrobial effects of methylene blue, helping to reduce microbial resistance and enabling new therapeutic applications.

## 4. Conclusions

The present study demonstrated that methylcellulose nanoparticles can effectively serve as carriers for methylene blue. Furthermore, by adjusting the synthesis conditions, the particle diameter can be fine-tuned to achieve controlled release, optimizing encapsulation efficiency. At a nanoparticle concentration of 25%, cell survival remained around 40%, indicating acceptable biocompatibility at lower concentrations. The antimicrobial activity of these nanoparticles surpassed that of free methylene blue, highlighting their enhanced effectiveness against a range of oral pathogens, including gram-positive bacteria, gram-negative bacteria, and fungi. The combination of photodynamic therapy (PDT) with encapsulated methylene blue nanoparticles represents an innovative and potent strategy for treating oral infections, with broad potential applications in various fields of dentistry.

## Figures and Tables

**Table 1:** Encapsulation efficiency, particle size, polydispersity index, and zeta potential of methylcellulose-loaded methylene blue nanoparticles.

Sample	Encapsulation Efficiency (%)	Particle Size (nm)	PDI	Zeta Potential (mV)
MC 0.001-MB 0.005	99.1 ± 0.1 ^a^	186.2 ± 3.1 ^d^	0.274 ± 0.110 ^c^	1.7 ±0.0 ^b^
MC 0.005-MB 0.005	98.8 ± 0.1 ^b^	274.1 ± 10.8 ^c^	0.353 ± 0.003 ^bc^	2.9 ± 0.3 ^ab^
MC 0.001-MB free	--	495.1 ± 7.0 ^a^	0.730 ± 0.007 ^a^	1.4 ± 0.4 ^b^
MC 0.005-MB free	--	314.0 ± 4.0 ^b^	0.665 ± 0.100 ^b^	6.6 ± 1.4 ^a^

(--): Not evaluated. Results expressed as mean ± standard deviation (*n* = 3). Different letters indicate a significant difference (*p* < 0.05) when analyzed by Tukey’s test in the column.

**Table 2:** Antimicrobial activity of methylene blue nanoparticles.

Sample	*S. mutans*	*A. actynomices*	*S. aureus*	*P. aeruginosa*	*E. coli*	*C. albicans*	*E. faecalis*
	CFU·mL^−1^						
MC 0.001-MB 0.005	3.5 × 10^3^ ± 7.1 × 10^2 c^	5 × 10^2^ ± 7.1 × 10^2 c^	10^3^ ± 0.0 ^b^	<10^2^	<10^2 z^	<10^2^	5 × 10^2^ ± 7.1 × 10^2 b^
MC 0.005-MB 0.005	<10^2^	5 × 10^2^ ± 7.1 × 10^2 b^	1.5 × 10^3^ ± 7.1 × 10^2 b^	5 × 10^2^ ± 7.1 × 10^2 c^	<10^2^	<10^2^	<10^2 c^
MC 0.001-MB free	1.5 × 10^3^ ± 7.1 × 10^2 b^	5 × 10^2^ ± 7.1 × 10^2 b^	5 × 10^2^ ± 7.1 × 10^2 b^	7 × 10^3^ + 03 ± 2.8 × 10^3 b^	<10^2^	<10^2^	<10^2 c^
MC 0.005-MB free	<10^2^	2.5 × 10^4^ ± 3.5 × 10^3 a^	1.5 × 10^3^ ± 7.1 x10^2 b^	1.5 × 10^3^ ± 7.1 × 10^2^	5 × 10^5^ ± 7.1 × 10^5 b^	<10^2^	<10^2 c^
MB 0.005	2.1 × 10^5^ ± 1.4 × 10^4 a^	2.3 × 10^4^ ± 3.5 × 10^3 a^	6.8 × 10^5^ ± 2.5 × 10^5 a^	1.3 × 10^5^ ± 2.1 x10^3 a^	2.2 × 10^8^ ± 3 × 10^8 a^	2.5 × 10^3^ ± 7.1 × 10^2 a^	4.9 × 10^5^ ± 1.7 × 10^4 a^

Different letters indicate a significant difference (*p* < 0.05) when analyzed by Tukey’s test in the column.

## Data Availability

The original contributions presented in this study are included in the article. Further inquiries can be directed to the corresponding author.
